# Complete and nearly complete genome sequences of seven strains of *Burkholderia glumae* and *B. gladioli* isolated from rice panicles in Vietnam

**DOI:** 10.1128/mra.00388-25

**Published:** 2025-06-18

**Authors:** Doan Thi Kieu Tien, Van Quoc Giang, Pham Van Luc, Kha Thi Tuong Vy, Huynh Thi Thong, Lam Van Hau, Pierre Lefeuvre, Nguyen Thi Thu Nga

**Affiliations:** 1 College of Agriculture, Can Tho University 595780 , Can Tho, Vietnam; 2 CIRAD, UMR PVBMT, College of Agriculture, Can Tho University, Can Tho, Vietnam; University of Strathclyde, Glasgow, United Kingdom

**Keywords:** *Burkholderia*, bacterial panicule blight, Vietnam

## Abstract

*Burkholderia gladioli* and *Burkholderia glumae* are two of the main pathogens associated with bacterial panicle blight, a major disease of rice worldwide. Here, we present complete and nearly complete genome assemblies of three *B. glumae* and four *B. gladioli* strains isolated from rice collected in the Mekong Delta area.

## ANNOUNCEMENT

Bacterial panicle blight (BPB) is a globally destructive disease of rice (1) primarily associated with *Burkholderia glumae* (2) and *Burkholderia gladioli* (3). In Vietnam, BPB is recognized as a constraint to rice production in all production areas (4, 5), but knowledge of the bacterial diversity in the country is limited to only 16S sequences of *B. glumae* and *B. gladioli* and a single draft genome of *B. glumae* available in the database.

In order to gain knowledge on the diversity of bacteria potentially associated with the disease in the country, panicles showing symptoms of BPB were sampled from seven rice fields in the Mekong Delta area from May to July 2024 (Table 1). Panicles were washed with 70% EtOH, cut into small pieces using scalpels, and ground with a glass rod in 50 µL of water. Products were streaked on King’s B medium plates (6) before incubation at 25°C for 2–3 days. Round colonies of 1–2 mm diameters that released yellow pigment were cultured in 5 mL King’s Medium B Broth for 24 h at 25°C and 120 rpm/min (titer of ~10^8^ CFU/mL) before genomic DNA extraction using the Monarch Spin gDNA Extraction Kit (NEB). Long-read Oxford Nanopore MinION sequencing was performed in multiplex on a FLO-MIN114 flow cell using the SQK-RBK114-24 kit for library preparation and following the manufacturer’s instructions. Default parameters were used for all software unless otherwise noted. Super-accurate basecalling of raw sequencing signal was performed using Dorado version 0.7.2 (https://github.com/nanoporetech/dorado) with a minimum mean read PHRED quality score cut off of 8 (Table 1). Reads were used for *de novo* assemblies with Canu version 2.2 (7), specifying a genome size of 6.5 Mbp. Contigs flagged as circular from Canu analysis were trimmed using genome coordinates defined by the software. After polishing the assemblies using Medaka version 2.0.0a2 (https://github.com/nanoporetech/medaka), the contigs were annotated using NCBI PGAP version 6.9 (8). Full-length 16S rRNA gene sequences were obtained from the PGAP annotation and were used to validate the *Burkholderia* genus classification using the RDP classifier version 2.14 and associated database (9). Further analysis using Mash version 2.1.1 (10) and the genome reference sequences classified in the *Burkholderia* genus as obtained from NCBI on 23 January 2025 confirmed that strains were isolates of *B. glumae* and *B. gladioli* (Table 1).

**TABLE 1:** Strain details and summary of assembly statistics

Strain	Collection date	Sampling location	Sampling latitude and longitude	SRA	Total reads (total Mb)	Reads *N*_50_	Assembly length (bp)	Species (most similar strain/1 − MASH distance)	Total No. of genes (No. of CDSs with protein)	Sequence accession number	Length (nt)	Coverage	Circular	Type	G + C content (%)
HG_1	11-06-2024	Vietnam: Hau Giang province	9.68880152 N 105.61437308 E	SRX27652183	57,968 (465.8 Mb)	19,202	8,231,763	*Burkholderia* *gladioli* (BBB-01/0.980)	7,339 (6,727)	CP183180.1	4,163,892	57	Yes	Chromosome	0.677
										CP183179.1	3,834,718	53	Yes	Chromosome	0.684
										CP183178.1	178,386	71	Yes	Plasmid	0.619
										CP183181.1	54,767	52	Yes	Plasmid	0.635
HG_2	11-06-2024	Vietnam: Hau Giang province	9.68880152 N 105.61437308 E	SRX27652184	14,399 (217.0 Mb)	30,003	8,231,327	*Burkholderia* *gladioli* (BBB-01/0.981)	7,271 (7,001)	CP183177.1	4,163,675	27	Yes	Chromosome	0.677
										CP183175.1	3,834,513	25	Yes	Chromosome	0.684
										CP183174.1	178,372	30	Yes	Plasmid	0.619
										CP183176.1	54,767	24	Yes	Plasmid	0.635
HG_3	11-06-2024	Vietnam: Hau Giang province	9.68880152 N 105.61437308 E	SRX27652187	116,451 (213.4 Mb)	3,366	6,824,497	*Burkholderia* *glumae* (ATCC 33617/0.993)	6,145 (5,602)	JBMJCS010000003.1	3,591,441	31	Yes	Chromosome	0.682
										JBMJCS010000008.1	74,873	29	No	Plasmid	0.613
										JBMJCS010000005.1	114,947	31	No	Plasmid	0.638
										JBMJCS010000004.1	12,382	21	No	Plasmid	0.604
										JBMJCS010000002.1	474,343	28	No	Chromosome	0.671
										JBMJCS010000006.1	59,376	24	Yes	Plasmid	0.589
										JBMJCS010000001.1	2,359,784	29	No	Chromosome	0.691
										JBMJCS010000007.1	137,351	36	Yes	Plasmid	0.613
HG_4	11-06-2024	Vietnam: Hau Giang province	9.68880152 N 105.61437308 E	SRX27652185	64,234 (610.0 Mb)	19,939	8,231,748	*Burkholderia* *gladioli* (BBB-01/0.980)	7,348 (6,758)	CP183173.1	4,163,896	75	Yes	Chromosome	0.677
										CP183170.1	3,834,690	70	Yes	Chromosome	0.684
										CP183172.1	178,381	75	Yes	Plasmid	0.619
										CP183171.1	54,781	58	Yes	Plasmid	0.635
HG_5	27-05-2024	Vietnam: Hau Giang province	9.96656307 N 105.59525536 E	SRX27652186	12,751 (206.5 Mb)	31,974	8,231,303	*Burkholderia* *gladioli* (BBB-01/0.981)	7,277 (7,019)	CP183166.1	4,163,677	27	Yes	Chromosome	0.677
										CP183168.1	3,834,485	22	Yes	Chromosome	0.684
										CP183169.1	178,373	30	Yes	Plasmid	0.619
										CP183167.1	54,768	26	Yes	Plasmid	0.635
HG_7	27-05-2024	Vietnam: Hau Giang province	9.96656307 N 105.59525536 E	SRX27652188	121,960 (345.7 Mb)	6,301	6,810,121	*Burkholderia* *glumae* (ATCC 33617/0.995)	6,108 (5,611)	JBMJCR010000001.1	2,890,753	38	Yes	Chromosome	0.686
										JBMJCR010000003.1	3,590,963	31	No	Chromosome	0.682
										JBMJCR010000005.1	129,652	28	No	Plasmid	0.615
										JBMJCR010000004.1	110,996	29	Yes	Plasmid	0.639
										JBMJCR010000002.1	87,757	38	No	Plasmid	0.615
LA_B1	20-07-2024	Vietnam: Long An province	10.74514622 N 105.69122389 E	SRX27652189	13,222 (104.4 Mb)	15,884	6,919,044	*Burkholderia* *glumae* (ATCC 33617/0.997)	6,234 (5,909)	JBMJCQ010000003.1	3,565,893	15	Yes	Chromosome	0.684
										JBMJCQ010000001.1	2,932,420	15	No	Chromosome	0.684
										JBMJCQ010000004.1	245,243	15	Yes	Plasmid	0.636
										JBMJCQ010000002.1	175,488	12	No	Plasmid	0.608

All *B. gladioli* assemblies comprised two closed circular chromosomal sequences. *B. glumae* strains presented with a single circular chromosomal sequence and one to two contigs that failed to be circularized for the other chromosome. One or two closed plasmids were obtained per strain (54,767–245,243 bp). Six additional uncircularized contigs of 12.4–175.5 kb were obtained and were assigned to plasmids based on online BLASTn (11) searches against the NCBI nr database. Totals of 6,108–7,348 genes were predicted, and completeness of the representative gene set was reported for all genomes using BUSCO version 5.8.0 (12). All strains possessed predicted tetracycline and benzalkonium chloride resistance genes carried on the chromosome, as detected using the online Resistance Gene Identifier server (13).

## Data Availability

The raw data are available under BioProject accession PRJNA1222096.
